# Aflatoxin B_1_ Degradation by *Stenotrophomonas Maltophilia* and Other Microbes Selected Using Coumarin Medium#

**DOI:** 10.3390/ijms9081489

**Published:** 2008-08-22

**Authors:** Shu Guan, Cheng Ji, Ting Zhou, Junxia Li, Qiugang Ma, Tiangui Niu

**Affiliations:** 1 National Key Laboratory of Animal Nutrition, China Agricultural University, Beijing 100094, China. E-Mails: guanshu8@gmail.com (S. G.); jicheng@cau.edu.cn (C. J.); maqiugang@cau.edu.cn (Q. M.); 2 Guelph Food Research Center, Agriculture and Agri-Food Canada, Guelph N1G 5C9, Canada. E-Mail: zhout@agr.gc.ca (T. Z.); 3 College of Food Science and Nutritional Engineering, China Agricultural University, Beijing 100083, China. E-Mail: lijunxia2003.88@163.com (J. L.)

**Keywords:** aflatoxin B_1_, degradation, culture supernatant, *Stenotrophomonas maltophilia*

## Abstract

Aflatoxin B_1_ (AFB_1_) is one of the most harmful mycotoxins in animal production and food industry. A safe, effective and environmentally sound detoxification method is needed for controlling this toxin. In this study, 65 samples were screened from various sources with vast microbial populations using a newly developed medium containing coumarin as the sole carbon source. Twenty five single-colony bacterial isolates showing AFB_1_ reduction activity in a liquid culture medium were selected from the screen. Isolate 35-3, obtained from tapir feces and identified to be *Stenotrophomonas maltophilia*, reduced AFB_1_ by 82.5% after incubation in the liquid medium at 37 °C for 72 h. The culture supernatant of isolate 35-3 was able to degrade AFB_1_ effectively, whereas the viable cells and cell extracts were far less effective. Factors influencing AFB_1_ degradation by the culture supernatant were investigated. Activity was reduced to 60.8% and 63.5% at 20 °C and 30 °C, respectively, from 78.7% at 37 °C. The highest degradation rate was 84.8% at pH 8 and the lowest was only 14.3% at pH 4.0. Ions Mg^2+^ and Cu^2+^ were activators for AFB_1_ degradation, however ion Zn^2+^ was a strong inhibitor. Treatments with proteinase K, proteinase K plus SDS and heating significantly reduced or eradicated the degradation activity of the culture supernatant. The results indicated that the degradation of AFB_1_ by *S. maltophilia* 35-3 was enzymatic and could have a great potential in industrial applications.

## 1. Introduction

Aflatoxins are a group of structurally related difuranocoumarin derivatives produced mainly by *Aspergillus flavus* and *Aspergillus parasiticus* [1]. Aflatoxin B_1_ (AFB_1_), one of the most hazardous mycotoxins, is extremely toxic, mutagenic and carcinogenic [2, 3]. It poses a severe threat to both livestock productivity and human health and thus, brings huge worldwide economic losses each year [4].

Various physical and chemical methods have been developed and tested for controlling AFB_1_. However, disadvantages of these methods, such as nutritional loss, sensory quality reduction and high cost of equipment, have limited their practical applications [5–9]. It is expected that progress in the control of mycotoxin contamination will depend on the introduction of technologies for specific, efficient, and environmentally sound detoxification. The utilization of microorganisms and/or their enzymatic products to detoxify mycotoxins in contaminated food and feed can be a choice of such technology [10, 11].

Recently, interests in biological detoxification of AFB_1_ have greatly increased. Several fungal species have been found to be able to transform AFB_1_ into less toxic metabolites; such fungi include *Pleurotus ostreatus* [12], *Trametes versicolor* [13], *Rhizopus* sp., *Mucor* sp.[14], and a few yeasts such as *Trichosporon mycotoxinivorans* [15], *Saccharomyces cerevisiae* [16], *Trichoderma* strains [17], and *Armillariella tabescens* [18]. The degradation activities of these fungi were mainly in their cell extracts. However, practical applications of these fungi may be limited by factors, such as long incubation time, e.g. more than 120 h, required for the detoxification and complicated procedures needed for obtaining the active extracts. Reduction of AFB_1_ by bacteria has also been reported; most of the published studies focused on lactic acid bacteria, such as strains belonging to *Lactobacillus* [19, 20], *Bifidobacterium* [21, 22], *Propionibacterium* [23] and *Lactococcus* [24]. However, the AFB_1_ reduction by these bacteria was proven to be mainly by cell binding rather than metabolism or degradation. Most importantly, this kind of binding seems to be reversible, which means that AFB_1_ can hardly be removed completely from contaminated media. Apart from this, bacteria effective in AFB_1_ degradation were limited to *Rhodococcus erythropolis* [25], *Mycobacterium fluoranthenivorans* [26, 27] and *Nocardia corynebacterioides* (formerly *Flavobacterium aurantiacum*) [28–31].

The current research is aimed at searching for new AFB_1_ degradation bacteria. An effective screening method was developed, which was used to screen for microbes capable of degrading AFB_1_ in samples collected from various natural sources. One of the obtained bacterial isolates, 35-3, exhibited strong degradation activity thus was further identified and characterized. Factors affecting degradation efficiency of the isolate were also investigated.

## 2. Results and Discussion

### 2.1. Screening for AFB_1_ degradation microbes

Twenty five single-colony bacterial isolates were obtained from 65 samples collected from various sources (Table 1). All these isolates were able to reduce concentrations of AFB_1_ in the liquid medium tested after 72 h incubation at 37 °C with various degrees of effectiveness. Sixteen isolates reduced AFB_1_ in the medium by over 50%. Isolate 35-3 was the most effective and reduced AFB_1_ by 82.5% (Table 1).

Volkl et al. [32] has proposed that biological degradation of mycotoxins occurs in nature since many mycotoxins are chemically stable but do not appear to accumulate in natural environments. Therefore, environmental samples rich in microorganisms, such as animal feces, decayed barks, soils and cereal grains, were chosen as sources for selection of microorganisms that degrade AFB_1_.

To identify active isolates from the vast microbial populations of environmental samples, an effective selection method is very much needed. In this study, a medium containing coumarin (CM) as the sole carbon source was developed for the first time and was used for the microbial selection. The microorganisms grew slowly and only very few colonies appeared on the medium. Single colonies were picked up after incubation of 3–7 days and transferred to fresh CM plates three times sequentially. Only 25 single colonies were selected out of huge populations with great diversities in the collected samples, and none was false positive. The results clearly indicated that this newly developed method was not only extremely selective but also accurate.

**Figure 1.**
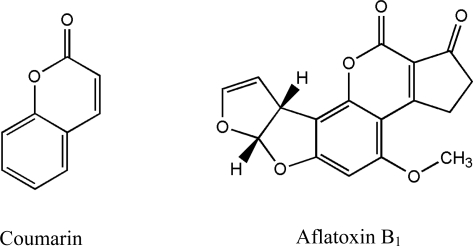
Molecular structures of coumarin and aflatoxin B_1_.

Aflatoxins are a group of bisfuranocoumarin derivatives and the lactone ring in the common coumarin structure plays an important role in its toxicity and mutagenicity [33]. Coumarin is the basic molecular structure of all aflatoxins (Figure 1) [34, 35]. Therefore, microorganisms that could utilize coumarin as their carbon source might also be able to use aflatoxins, in this case, AFB_1_. The metabolizing processes should result in degradation of the mycotoxin. Coumarin is a phytochemical, which is widely used in flavor industry for sour. Compared with AFB_1_, it is much safer for users, easier to obtain and cheaper to buy. The developed coumarin method provided an inexpensive, feasible and effective tool for selecting AFB_1_ degradation microorganisms. The method should also be useful in research targeting other aflatoxins.

### 2.2. Identification of isolate 35-3

**Figure 2.**
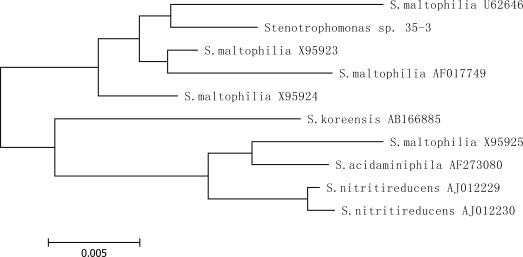
Phylogenetic tree based on 16S rRNA gene sequences of isolate 35-3 and related taxa.

Isolate 35-3 appeared on nutrient agar as round straw yellow colored colonies. It is a gram-negative bacterium. The isolate grew well at 37 °C, but not at 10 °C or 55 °C. It was able to use most single sugars including glucose, maltose and sucrose as a sole carbon source. The isolate could hydrolyze gelatin and Tween 80 but not amylum (Table 2). Determination of the 16S rRNA gene sequence revealed that the isolate belonged to genus *Stenotrophomonas* (Figure 2). The closest relationship (99% sequence similarity) obtained with the type of a described species was *Stenotrophomonas maltophilia* U62646, which is an aerobic gram-negative bacterium. It has been reported that isolates from this genus possess function in degradation of polycyclic aromatic hydrocarbons (PAHs) and enzymes are involved in these processes [36–38]. However, this is the first report indicating that a bacterium in this genus possesses function in mycotoxin degradation.

### 2.3. AFB_1_ degradation by S. maltophilia 35-3

**Figure 3.**
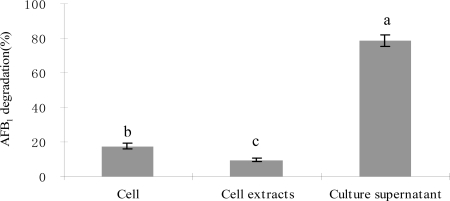
AFB_1_ degradation by cell, cell extracts and culture supernatant of *S. maltophilia* 35-3 after 72 h incubation. The values are means of three replicates and their standard errors. Means with different letters are significantly different according to Duncan’s Multiple Range Test (P <0.05).

Culture supernatant of *S. maltophilia* 35-3 showed strong AFB_1_ degrading activity and it was more effective (P<0.05) than viable cells and cell extracts (Figure 3). Culture supernatant was able to degrade 78.7% AFB_1_ after 72 h incubation compared to 17.5% and 9.6% by viable cells and cell extracts, respectively.

Culture supernatant of *Rhodococcus erythropolis* degraded AFB_1_ with 33.2% residual after 72 h incubation, and the degradation was proved to be enzymatic by using proteinase K and SDS treatments [25]. Similarly, the original culture supernatant of *Flavobacterium aurantiacum* degraded 74.5% of AFB_1_ in 24 h and it could only degrade 34.5% of AFB_1_ after being treated with proteinase K (0.1 mg/mL) [28]. The active ingredient in the culture supernatant was considered to be a protein or perhaps an enzyme [25, 28]. In this study, the activity of AFB_1_ degradation was mainly in the culture supernatant of *S. maltophilia* 35-3 rather than its cells or cell extracts. Degradation of AFB_1_ by the culture supernatant produced without pre-exposure to AFB_1_ indicated that the degradation was achieved during the normal growth of the bacterium, suggesting that the degradation was a constitutive activity of *S. maltophilia* 35-3. Culture supernatant treated with proteinase K displayed significantly reduced degradation ability (23.8%). When culture supernatant was treated with proteinase K plus SDS and heat (boiling water bath for 10 min), respectively, no degradation activity was observed. All these results implied that a protein or enzyme might be involved in the degradation by *S. maltophilia* 35-3.

**Figure 4.**
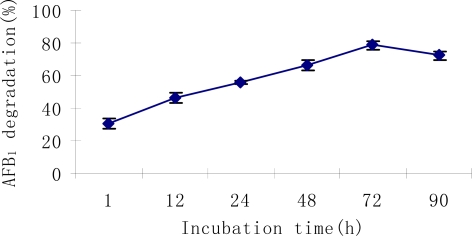
Dynamics of AFB_1_ degradation by *S. maltophilia* 35-3 culture supernatant with time.

Degradation of AFB_1_ by the culture supernatant of *S. maltophilia* 35-3 was a relatively rapid and continues process, with 46.3% AFB_1_ degraded in the first 12 h and 78.7% degraded after 72 h (Figure 4). Similar results were obtained elsewhere. Alberts et al. [25] reported a 66.8% reduction of AFB_1_ from 0 to 72 h when incubated with culture supernatant of *R. erythropolis*; Hormisch et al. [26] indicated that liquid cultures of *Mycobacterium* strain FA4 could reduce AFB_1_ level by 70 to 80% within 36 h and completely degrade AFB_1_ in 72 h. In comparison, most of the lactic acid bacteria that were able to bind AFB_1_ could rapidly remove the toxin from liquid; however, they would release AFB_1_ to some extent in the prolonged incubation period [19, 22]. The continuous increase in detoxification by *S. maltophilia* 35-3 with time indicated that binding might not play important role in the AFB_1_ reduction.

**Figure 5.**
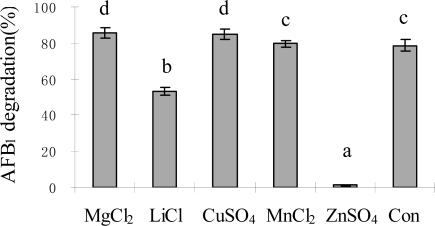
Effects of ions on AFB_1_ degradation by culture supernatant of *S. maltophilia* 35-3. Nutrient broth (NB) was used to substitute culture supernatant as a control. The values are means of three replicates and their standard errors. Means with different letters are significantly different according to Duncan’s Multiple Range Test (P <0.05).

AFB_1_ degradation was strongly affected by metal ions (Figure 5). Ions Mg^2+^ and Cu^2+^ showed effect on stimulating AFB_1_ degradation at the concentration of 10 mM compared with control (78.7%). Their degradation rates were 85.4% and 85.0%, respectively. However, Li^+^ ions at 10 mM reduced the degradation to 53.3% and Zn^2+^ ions inhibited the activity even more significantly, with only 1.4% of AFB_1_ degraded after 72 h. These results agreed with studies of D'Souza and Brackett [29] in effects of Mg^2+^ on AFB_1_ degradation by *F. aurantiacum*. Additions of 0.1, 1 and 10 mM Mg^2+^ increased AFB_1_ degradation after 48 h incubation. The explanation could be that Mg^2+^ might stabilize membranes, maintain structural integrity of proteins and act as enzyme activator. Also, AFB_1_ degradation by *F. aurantiacum* was significantly inhibited (P<0.05) after incubation with 10 mM Zn^2+^ for 4, 24 and 48 h [30], which was similar to that was noticed in our study. Zn^2+^ might be able to alter the enzyme system by causing a conformational change in the enzymes to a form with lower affinity for AFB_1_ degradation, or by inactivating the enzyme [30]. The effects of ions on activity of *S. maltophilia* 35-3 further supported the enzyme involvement in AFB_1_ degradation by the isolate.

**Figure 6.**
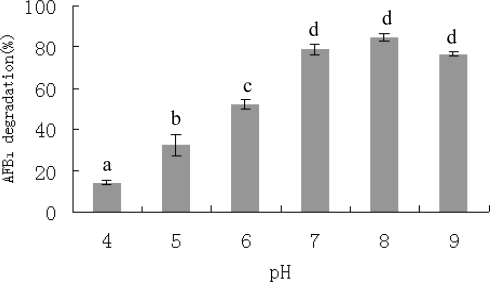
Effect of pH on AFB_1_ degradation by culture supernatant of *S. maltophilia* 35-3. The values are means of three replicates and their standard errors. Means with different letters are significantly different according to Duncan’s Multiple Range Test (P <0.05).

AFB_1_ degradation by the culture supernatant was pH sensitive (Figure 6). The highest degradation (84.8%) was observed at pH 8.0 and it decreased gradually as the pH value went down, to the lowest at pH 4.0 (14.3%). However, the culture supernatant maintained its degradation ability (76.9%) in basic condition at pH 9.0. No degradation was detected in controls with different pH values. The effect of pH on degradation of AFB_1_ by cell extracts of *F. aurantiacum* showed a similar trend [31]. The degradation of AFB_1_ was approximately 25% at pH 5, increased to 50% at pH 6 and 70% at pH 7, and decreased to 50% at pH 8. The correlation of AFB_1_ degradation with pH values is typical for enzymatic reactions. Enzymes have an optimal pH range for maximal activities. At pH values outside of the optimum, enzymatic activity decreases due to the ionization of a critical amino acid residue within the catalytic site [39]. The maximal AFB_1_ degradation by *S. maltophilia* 35-3 in this study was observed at a basic pH (pH 8), indicating the enzyme produced by the isolate had a higher optimal pH compared to enzyme in cell extracts of *F. aurantiacum* [31].

**Figure 7.**
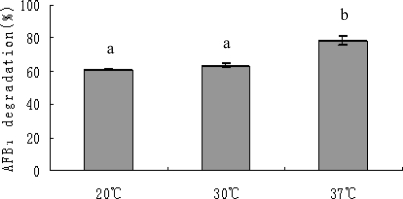
Effect of temperature on AFB_1_ degradation by culture supernatant of *S. maltophilia* 35-3. The values are means of three replicates and their standard errors. Means with different letters are significantly different according to Duncan’s Multiple Range Test (P <0.05).

The AFB_1_ degradation by *S. maltophilia* 35-3 culture supernatant varied under different temperatures (Figure 7). The degradation was lower at 20 °C (60.8%) and 30 °C (63.5%) than at 37 °C (78.7%) (P<0.05). The isolate 35-3 was originated from feces of South American tapir; temperature at 37 °C should be more suitable for the survival and growth of the bacterium, thus optimal for its enzyme system. Teniola et al. [27] reported that AFB_1_ degradation by cell extracts of *R. erythropolis* and *M. fluoranthenivorans* were about the same in between 10–40 °C (> 90%). They proposed either that the enzymes in the extracts had a wide temperature range of activity or that other factors were involved in the degradation.

## 3. Experimental Section

### 3.1. Culture media

Each liter of coumarin medium (CM) contained 10.0 g coumarin (Beijing Chemical Inc., China), 0.25 g KH_2_PO_4_, 1.0 g NH_4_NO_3_, 1.0 g CaCl_2_, 0.25 g MgSO_4_.7H_2_O, 1.0 mg FeSO_4_, and 15.0 g agar. The pH of the medium was adjusted to 7.0. Nutrient broth (NB) consisted of 3.0 g yeast extract, 5.0 g peptone, 6.0 g glucose, 10.0 g NaCl per liter (pH=7.0). Nutrient agar (NA), which was NB plus 15 g agar, was used for preserving microbial isolates.

### 3.2. Isolation of microorganisms

#### 3.2.1. Samples

Sixty-five samples were screened for AFB_1_ degradation activity. The samples consisted of thirty nine feces of wild animals collected from Beijing Zoo, Beijing, China; nineteen cereal grains, obtained from Beijing Huilongguan foodstuff market; five soil samples and two decayed tree bark samples collected from farmland in China Agricultural University, Beijing, China. All these samples were air-dried at room temperature.

#### 3.2.2. Isolation

Samples (0.5 g) were ground with 5.0×10^5^ IU nystatin (Beijing Chemical Inc., China) before being homogenized in sterilized distilled water (9.0 mL). After incubation at room temperature on a rotary shaker for 12 h, the supernatant was serially diluted with sterilized distilled water to 10^−1^, 10^−2^, 10^−3^, 10^−4^, 10^−5^ folds. Diluted aliquots (0.2 mL) were plated on plates of CM medium, which were incubated at 37 °C for 3–7 days until visible colonies appeared. Single colonies were isolated and subsequencially transferred to fresh CM plates for three times. Colonies that were able to grow on the medium were selected and preserved as pure isolates on NA, and tested for AFB_1_ degradation.

### 3.3. Tests of AFB_1_ degradation

Degradation of AFB_1_ by the selected isolates was carried out in liquid cultures. The microbial isolates were cultured in NB. For inoculation, 12 h culture broth (2.5 mL) was transferred to NB (50 mL) in a 300 mL flask. The microbes were grown at 37 °C with agitation at 140 g for 24 h in a Gyrotary shaker incubator (Haerbin Donglian Electronic Equipment Inc., China). AFB_1_ standard solution (Sigma Chemical Co., Bellefonte, USA) was diluted with methanol (Beijing Chemical Inc., Beijing, China) to a stock solution of 500 ppb, of which 0.2 mL was added to microbial cultures of 0.8 mL for a final concentration of 100 ppb. The degradation tests were conducted in the dark at 37 °C without shaking for 72 h. After incubation, cells of microbes were removed by centrifugation at 10,000 g for 10 min (Beijing Medical Centrifugator Inc., China). Sterile NB was used to substitute microbial culture in the control.

For AFB_1_ analysis, the HPLC procedure by AOAC [40] was used with slight modifications. The reaction mixtures were extracted three times with chloroform. The chloroform extracts were evaporated under nitrogen at room temperature, the residue were dissolved in 50% methanol in water (1:1, v/v) and analyzed by HPLC. HPLC analysis was performed using a LiChroCART RP-C18 (250-4 Hypersil ODS (5 μm), Merck) column with a guard column (LiChroCART 4-4 RP-C18 (5 μm), Merck). The mobile phase was methanol: water (1:1, v/v) isocratic at a flow rate of 1 mL/min. AFB_1_ was derived by a photochemical reactor (AURA, USA) and measured by a fluorescence detector. The excitation and detection wavelengths were set at 360 and 440 nm, respectively. The percentage of AFB_1_ degradation was calculated using the following formula:

$$(1{-\text{AFB}}_{1}\;{\text{peak area in treatment }/\text{ AFB}}_{1}\;\text{peak}\;\text{area in control})\;\times \;100\%$$

### 3.4. Characterization of S.maltophilia 35-3

#### 3.4.1. Physiological and biochemical tests

Physiological and biochemical tests were carried out following the method of Holt *et al*. [41].

#### 3.4.2. Determination of 16S rRNA gene sequence

DNA extraction was done by using TIANamp Bacterial DNA Kit (Beijing TIANGen Biotech, China) according to the manufacturer’s instructions. PCR–mediated amplification of the 16S rDNA, purification and sequencing of the PCR products were done by Beijing Genomics Institute. The primers used for amplifying and sequencing were: 27f (5’-GAGAGTTTGATCCTGGCTCAG-3’), 530f (5’-GTGCCAGCAGCC GCGG-3’) and 1541r (5’-AAGGAGGTGATCCAGCCGCA-3’).

#### 3.4.3. Phylogenetic analyses

The generated DNA sequences and sequences derived from GenBank were aligned using the ClustalX program [42]. Neighbour joining analysis and calculation of bootstrap values were done according to the MEGA program [43].

### 3.5. Degradation of AFB_1_ by S. maltophilia 35-3

*Stenotrophomonas maltophilia* isolate 35-3 was selected for further study owing to its high degradation efficiency. Unless specifically indicated, all degradation experiments were conducted under 37 °C for 72 h with aeration.

#### 3.5.1. Degradation of AFB_1_ by *S. maltophilia* 35-3 cells

Fresh NB was inoculated with 12 h pre-cultured isolate 35-3 at 37 °C, agitation at 140 g for 24 h in a Gyrotary shaker incubator. Cells were pelleted using a refrigerated high-speed centrifuge (GL-20G-II centrifugator, Shanghai Anting Instrument Inc., China) at 10,000 g, 4 °C for 10 min. The pellets were washed twice with phosphate buffer (50 mM; pH 7.0) before resuspension in the phosphate buffer (5 mL) [19]. The AFB_1_ degradation tests were performed as described in 3.3. Phosphate buffer was used to substitute bacterial cell suspensions in the control samples.

#### 3.5.2. Degradation of AFB_1_ by *S. maltophilia* 35-3 intracellular cell extracts

Cell pellets were prepared as described previously (3.5.1). Pellets were suspended in phosphate buffer (pH 7.0; 3 mL buffer per gram cell mass). The suspension was disintegrated twice (work every other 5 s for 33 min) by using ultrasonic cell disintegrator on ice (Ningbo Xinzhi Instruments Inc., China). The disintegrated cell suspension was centrifuged at 12,000 g for 20 min at 4 °C. The cell extracts were collected by filtering the supernatant aseptically using 0.2 μm pore size sterile cellulose pyrogen free filters (Beijing Biotech Inc., China). The AFB_1_ degradation tests were performed as described in 3.3. Phosphate buffer solution was used to substitute intracellular cell extracts in the control.

#### 3.5.3. Effects of incubation period, temperature, pH, metal ions and proteinase K treatment on AFB_1_ degradation by *S. maltophilia* 35-3 supernatant

Isolate 35-3 grown in NB for 24 h was centrifuged with 10,000 g at 4 °C for 20 min, and the resulting culture supernatant was tested for AFB_1_ degradation. AFB_1_ methanol stock solution (0.2 mL) was added to 0.8 mL culture supernatant in a 7 mL tube. The reaction mixture was incubated in the dark at 37 °C without shaking for 1, 12, 24, 48, 72 and 90 h, respectively. To determine the effect of temperature, the mixtures were incubated at 20, 30 and 37 °C, respectively for 72 h. Controls were set at the above temperatures by using NB medium.

In the pH tests, initial pH value was obtained by adjusting pH to 4.0, 5.0 and 6.0 with citrate acid buffer, and to 7.0, 8.0 and 9.0 by sodium phosphate buffer. Controls were set by adjusting NB medium to different pH values.

The effects of different metal ions on degradation were determined by adding Mg^2+^, Zn^2+^, Cu^2+^, Mn^2+^ and Li^+^ (in the form of MgCl_2_, ZnSO_4_, CuSO_4_, MnCl_2_ and LiCl, respectively) to the reaction mixture respectively resulting in a final ion concentration of 10 mM. NB was used to substitute culture supernatant in the control.

The effect of protease treatment was determined by exposing the culture supernatant to 1 mg/mL proteinase K (Roche Diagnostics, Basel, Switzerland; specific activity ≥30 U/mg) for 1 h at 37°C; 1 mg/mL proteinase K plus 1% SDS for 6 h at 37 °C. The effect of heat treatment was determined by dipping the culture supernatant in boiling water bath for 10 min. The untreated culture supernatant was used as control.

### 3.6. Statistical analyses

Data was analyzed as a completely randomized single factor design by ANOVA using the general linear models procedure in SAS. Significant F tests at the 0.05 levels of probability are reported. When a significant F-value was detected, Duncan’s Multiple Range Test was used to determine significant differences among means.

## 4. Conclusions

An innovative method with coumarin as a selective agent was developed and used to search for AFB_1_ degradation microorganisms in this study. The results have proven that the method is effective and accurate; the method is also safe, practical and economical. Twenty-five purified isolates were obtained using this method and all were able to degrade AFB_1_. Isolate 35-3, a bacterium belonging to *Stenotrophomonas maltophilia*, was identified for the first time to have the function of degrading AFB_1_. Enzyme(s) in the culture supernatant of the isolate might be responsible for the degradation although further confirmation is needed. Research is underway to purify the effective enzyme(s) and to identify metabolites produced during the degradation processes. The AFB_1_ degradation enzymes, once identified, may be mass-produced by the bacterial isolates and used to treat materials contaminated with AFB_1_. Furthermore, identification of genes responsible for AFB_1_ degradation can provide potential for AFB_1_ control with genetically modified microbes and crop cultivars.

## Figures and Tables

**Table 1.** AFB_1_ degradation by individual microbial isolates selected using coumarin medium.

Isolate^1^	Source	Degradation (%) ± SE^2^
*Stenotrophomonas maltophilia* (35-3)	South American tapir feces	82.50 ± 3.20^a^
*Bacillus* sp.	Hog deer feces	80.93 ± 2.65^ab^
*Brevundimonas* sp.	Yellow cheek feces	78.10 ± 4.48^bc^
*Bacillus* sp.	Farm soil	77.80 ± 1.63^bcd^
*Klebsiella* sp.	Rabbit feces	77.57 ± 4.36^cd^
*Brevundimonas* sp.	Goral feces	76.83 ± 0.72^cd^
*Enterobacter* sp.	Hog deer feces	75.92 ± 3.44^cd^
*Brachybacterium* sp.	Rabbit feces	74.83 ± 2.47^cd^
*Rhodococcus* sp.	Ostrich feces	73.92 ± 5.48^cd^
*Cellulosimicrobium* sp.	Farm soil	73.75 ± 3.60^d^
32-2	Goral feces	67.64 ± 1.72^e^
K2	Deer feces	67.64 ± 0.75^e^
41-4	Zebra feces	64.81 ± 4.84^e^
K3	Deer feces	64.23 ± 1.44^e^
I1	Francois monkey feces	58.76 ± 2.48^f^
N1	Farm soil	51.50 ± 0.57^g^
23-5	Goral feces	48.69 ± 3.18^gh^
G3	Zebra feces	46.39 ± 1.25^h^
42-1	Compound feed	45.18 ± 1.30^h^
J1	Red goral feces	30.88 ± 2.82^i^
39-3	White cheek feces	28.08 ± 1.25^i^
37-1	Leopard feces	18.71 ± 0.87^j^
H1	Farm soil	13.94 ± 1.01^k^
31-3	Compound feed	11.91 ± 2.01^k^
C1	Grey leaf monkey feces	9.18 ± 1.54^k^

1 AFB_1_ degradation in liquid medium following 72 h of incubation with individual microbial isolates appeared on medium with coumarin as the sole carbon source.

2 The values are means of three replicates and their standard errors. Means with different letters are significantly different according to Duncan’s Multiple Range Test (P <0.05).

**Table 2.** Biochemical and physiological characteristics of *Stenotrophomonas maltophilia* 35-3.

Item	Result^1^	Item	Result^1^	Item	Result^1^
**Carbon utilization:**		L-Glutamic acid	+	Casein	+
Glucose	+	**Nitrogen utilization:**		Oxidase	−
D(+)-Cellobiose	+	Ammonium oxalate	−	**Degradation of:**	
Sorbitol	w	(NH_4_)_2_SO_4_	−	Sodium alga acid	−
L- Arginine	−	NH_4_H_2_PO_4_	−	Cellulose	−
L-Phenylalanine	−	Glutamic acid	−	lignan xylan	−
Maltose	+	Proline	−	Lecithin	−
Mannitol	+	NaNO_2_	−	Yeast cell	+
D- Fructose	+	NH_4_NO_4_	+	**Utilisation of acid:**	
Galactose	+	Ammonium citrate	−	Citric acid	+
Amylum	+	**Growth at:** 10 °C/ 55 °C	−	Benzoic acid	+
D-Raffinose	+	**Growth on:**		Tartaric acid	+
Mannose	w	0% / 2% NaCl	+	Succinic acid	+
Glycine	+	5% / 7% / 10% NaCl	−	Acetic acid	−
L- Cysteine	−	**Hydrolysis of:**		**Other tests:**	
L-Tyrosine	−	Gelatin	+	Congo red tolerance	+
D- Xylose	+	Olein	−	V-P test	−
Sucrose	+	Tween 80	+	Methyl red test	−
A-Lactose	+	Amylum	−	Methylene blue trihydrate reduction	+

1 ‘+’ positive response; ‘−’ negative response; ‘w’ weak positive response.
